# Silencing of Carboxypeptidase E expression inhibits proliferation and invasion of Panc-1 pancreatic cancer cells

**DOI:** 10.12688/f1000research.53737.2

**Published:** 2022-04-28

**Authors:** Hong Lou, Y Peng Loh

**Affiliations:** 1 Section Cellular Neurobiology, National Institute of Child Health and Human Development of the National Institutes of Health, Bethesda, MD, 20892, USA

**Keywords:** Carboxypeptidase E, pancreatic cancer, cell proliferation, cell migration, cell invasion

## Abstract

**Background: **Pancreatic cancer is one of the leading cause of cancer-related death globally. The molecular basis of this disease is complex and not fully understood. Previous studies have indicated that carboxypeptidase E (CPE) plays a role in promoting tumorigenesis in many cancer types. Here we have investigated the effect of carboxypeptidase E (CPE), including its isoform, in regulating the proliferation, migration and invasion of Panc-1 cells, a pancreatic cell line.

**Methods:** Panc-1 cells were transfected with CPE siRNA which targets both CPE-wild type and its isoform, or scrambled siRNA, for 24 h and then assayed for proliferation by the MTT and colony formation assays, and migration and invasion by wound healing and matrigel assays, respectively.

**Results: **CPE siRNA treatment of Panc-1 cells down-regulated the expression of CPE mRNA by 94.8%. Silencing of CPE mRNA expression resulted in a significant decrease in proliferation as revealed by the MTT assay and a 62.8% decrease in colony formation. Western blot analysis of expression of Cyclin D1 in Panc-1 cells treated with CPE siRNA showed a decrease of 32.5% compared to scr siRNA treated cells, indicating that CPE regulates proliferation through modulating this cell cycle protein.  Additionally, suppression of CPE expression in Panc-1 cells significantly decreased migration and invasion.

**Conclusions: **Our findings indicate that CPE may play an important role in regulating cell proliferation, migration and invasion to promote pancreatic cancer tumorigenesis.

## Abbreviations

CPE, carboxypeptidase E

HCC, hepatocellular carcinoma

WT, wild type

## 1. Introduction

Pancreatic cancer is an intractable malignancy which has one of the highest mortality rates world -wide. Patients with pancreatic cancer seldom display any symptoms until an advanced stage. It is one of the most lethal malignant carcinomas, with a survival rate of 9% over five years. The incidence of pancreatic cancer is projected to increase worldwide, despite the availability of better methods for early diagnosis. Generally, surgery, chemotherapy, and radiotherapy are used to prolong survival, but there is no cure for advanced stage patients. ^1^ Hence, it is necessary to search for new molecular targets in order to develop novel therapeutic approaches to treating pancreatic cancer.

Carboxypeptidase E (CPE) is an enzyme that processes prohormones ^2, 3^ and has also been shown to be a trophic factor mediating neuroprotection, stem cell differentiation, and the regulation of bone mass, independent of its enzymatic activity. ^4– 6^ CPE is expressed in the brain and endocrine organs, ^7^ but also in epithelial-derived cancers such as colorectal, liver, cervical, and lung cancers. ^8– 11^ Clinical investigations have revealed that elevated CPE levels are correlated with poor prognosis in colorectal, cervical, and liver cancers, as well as lung adenomas. ^8, 9, 11, 12^ Additionally, CPE is a tumor survival factor under hypoxic conditions. ^13^ Recently, two major forms of CPE have been cloned and identified in human hepatocellular carcinoma (HCC) cell line: wild-type CPE (50-53 kD in size) and an N-terminal truncated splice variant named 40 kD CPE-ΔN. ^10^

In the present study, we investigated the effect of silencing the expression of CPE by siRNA on proliferation, migration, and invasion in Panc-1 cells. We also studied the mechanism by which CPE regulates Panc-1 cell proliferation.

## 2. Methods

### 2.1 Cell lines

The human pancreatic cell line Panc-1 was purchased from ATCC (Manassas, VA, RRID:CVCL_0480). The cells were cultured in DMEM media (Millipore Sigma, Burlington, MA, USA), supplemented with 10% fetal bovine serum (Thermo Fisher Scientific, Waltham, MA, USA) at 37 °C in a humidified 5% CO _2_ incubator.

### 2.2 Western Blot of cell lysates

Proteins from cells were extracted with RIPA lysis and extraction buffer (Thermo Fisher, Waltham, MA) supplemented with Complete Inhibitor Cocktail (Roche Applied Science, Indianapolis, IN). 20 μg of the protein from cell lysate was loaded per lane on SDS-PAGE gel and subjected to Western blotting according to our procedure published previously. ^13^ Antibodies against GAPDH (1:5000 dilution) and anti-cyclin D1 (1: 500 dilution) were purchased from Cell Signaling Technology (Danvers, MA).

### 2.3 SiRNA treatment

Panc-1 cells plated at 40–50% density were grown to ~75% confluency overnight as described above. The next day, cells were treated with 50-60 pmols of three CPE siRNAs custom synthesized by Invitrogen, (Carlsbad, CA) which target both CPE-WT and CPE-ΔN mRNAs:

siCPE Seq1#: GAU UUG UCC GAG ACC UUC AAG GUA A;

siCPE Seq2#: UUA CCU UGA AGG UCU CGG ACA AAU C;

siCPE Seq3#: GAU CCU GAG AGU UCC GAA CGU UUA A,

or scrambled siRNA, which is a non-targeting 20-25nt siRNA (Santa Cruz Biotechnology, Dallas, TX), using Lipofectamine RNAiMAX transfection reagent (Invitrogen, Carlsbad, CA) according to manufacturers’ protocol. After 24h incubation, the cells were dissociated by trypsinization, cell count were obtained, and seeded in various plates for cell invasion, cell migration, colony formation assay, and cell proliferation assays.

### 2.4 Quantitative real-time RT-PCR

Panc-1 cells were treated for 48h with siRNA. RNA was then extracted from the cells using the RNeasy Mini Kit (Qiagen, Germantown, MD). First-strand cDNA was synthesized with 200 ng of total RNA using the SensiFAST cDNA Synthesis Kit (Bioline Reagents Ltd, United Kingdom) for assay of CPE expression. Quantitative PCR was performed using 1μl first strand cDNA and SYBR Green Master Mix (Invitrogen) under the conditions of 95 °C for 15 s, annealing at 62 °C for 60 s, extension at 72 °C for 30 s for 40 cycles, and a final extension at 72 °C for 10 min. 18 s rRNA was used as a normalizing control. Primer sequences were: for amplifying CPE (generic), fwd: 5′- CCATCTCCGTGGAAGGAATA and rev: 5′-CTT ACA GCC TCA GCT CCA GG; 18S RNA, fwd: 5′-CTCTTAGCTGAGTGTCCCGC and rev: 5′-CTGATCGTCTTCGAACCTCC.

### 2.5 MTT assay

Panc-1 cells treated with siRNA for 24 h were seeded in a 96-well plate at a density of 2000 cells/well in 200μl media and incubated for four to five days. MTT (3-(4,5-dimethylthiazol-2-yl)-2,5-diphenyltetrazolium bromide) assay was performed daily from day one to four. 25 μl of MTT reagent (5 mg/ml) (Sigma-Aldrich, St. Louis, MO) added to each well were then incubated in a CO _2_ incubator at 37°C for 4 h. The supernatant was then removed and DMSO (150 μl) was added to each well. Absorbance value at 490 nm was then measured in a microplate reader (BioTek, Winooski, VT) after 5 min. Experiments were performed in five different wells for each condition. Two independent experiments were performed on separate days.

### 2.6 Colony formation assay

Panc-1 cells treated with CPE siRNA or scrambled siRNA were trypsinized and single cell suspensions were obtained. Viable cells, (2000/well) were seeded in a 6-well plate and cultured for 15-21 days. Culture medium was changed every five days. Cells were then gently washed twice with PBS and fixed in 100% methanol, before staining with 1% crystal violet solution for 10 minutes. Excess stain was removed by washing with PBS. The number of colonies containing at least 50 cells were counted with Image J software from the images captured under a light microscope. The experiment was repeated three times and each experiment was done in triplicates.

### 2.7 Wound healing assay

Approximately 1 × 10 ^5^ Panc-1cells were plated in the well of culture insert in non-coated 35 mm culture dish (Ibidi, Martinsried, Germany) and allowed to form a monolayer. A ~500 μm wound gap was created when the culture insert was removed, and the cells were immersed in the complete media following manufacturer’s instructions. Images of the wound were captured at time 0, and then at 12, 24, and 36 h after incubation in a CO _2_ incubator at 37°C. Wound closure was evaluated by measuring the areas of the wound at different time points using Image J software. Three experiments were done on separate days and each experiment was done in triplicates.

### 2.8 Invasion assay

A 24-well Corning Matrigel invasion chamber (Corning, NY) with 8-micron pores was used for cell invasion assay. 500 μl of cell suspension (1 × 10 ^5^ cells/ml) in serum free media was added to the top chamber. Then 500 μl of FBS supplemented media was added to the lower chamber to serve as a chemoattractant. 24 h. later, cells that did not invade through the pores were carefully removed with a cotton swab. Cells on the lower surface of the membrane were fixed with 100% methanol and stained with 1% crystal violet solution for 10 min. Excess stain was removed with water and images from five different fields/well were captured. Cells were counted with ImageJ software. Experiments were done in triplicates.

### 2.9 Statistical analysis

The data reported are the mean ± SD (standard deviation) or mean ± SE (standard error) of at least triplicate values (n) in each experiment. Number of independent experiments (N) is stated in the figure legends. Statistical significance was determined by Student’s t-test and p values are indicated as *p < 0.05, **p < 0.01, ***p < 0.001.

## 3. Results

### 3.1 Down-regulation of CPE expression in Panc-1 cells inhibits proliferation via Cyclin D1

**Figure 1.**
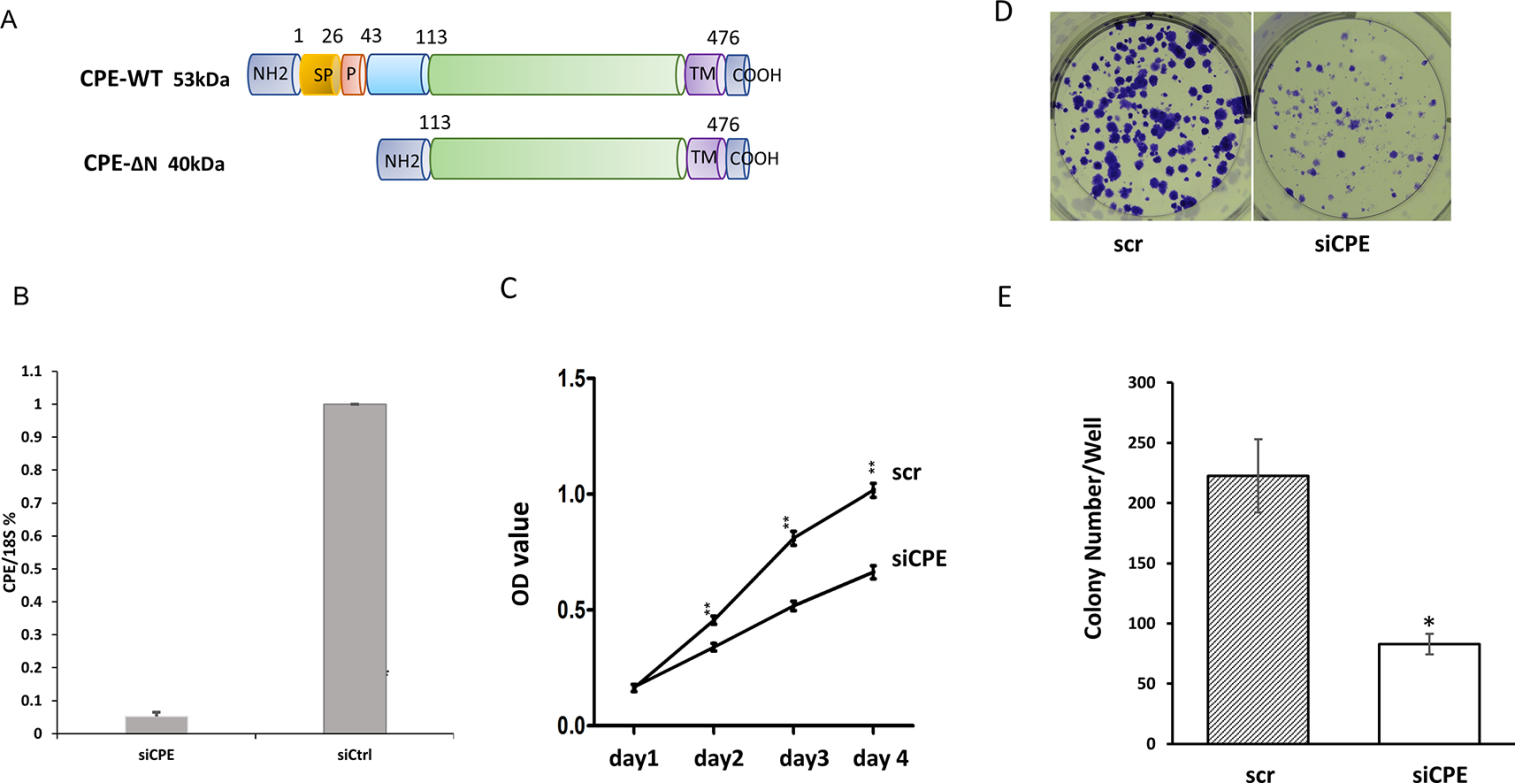
CPE siRNA treatment decreased proliferation of Panc-1 cells. A. Schematic showing the structure of 53 kDa CPE-WT and 40 kDa CPE-ΔN protein. SP, signal peptide; P, pro-region; TM, transmembrane domain. B. Bar graph showing CPE mRNA expression detected by qRT-PCR in Panc-1 cells treated with CPE siRNA (siCPE) or scrambled siRNA (scr). (**p < 0.01, N = 4). Error bars denote SD. C. Line graphs of MTT assay data showing the time course of cell proliferation of Panc-1 cells treated with CPE siRNA or scr siRNA, (day 2 - day4: **p < 0.01, n = 5). This is representative of two independent experiments with similar results. Error bars denote SEM. D. Image of colony formation assay of Panc-1 cells treated with CPE siRNA or scr si RNA. E. Bar graphs showing decrease in number of colonies/well formed after CPE siRNA (siCPE) treatment compared with scr siRNA controls (*p < 0.05, N = 3). Error bars denote SEM.

Figure 1A shows a schematic of the two forms of CPE: wild type CPE and 40 kD CPE-ΔN expressed by Panc-1 cells reported previously. ^14^ Moreover, CPE-WT protein was detected mainly in the secretion medium, while CPE-ΔN was found in the nucleus and cytoplasm of Panc-1 cells. ^14^ To evaluate the function of CPE, Panc-1 cells were treated with CPE si-RNA targeting the silencing of expression of both forms of CPE (see materials and methods). Figure 1B shows that treatment of Panc-1 cells with CPE siRNA down-regulated expression of CPE-transcripts by 94.8 ± 2.2%. Down-regulation of CPE with siRNA treatment resulted in significant inhibition of Panc-1 cell proliferation from day two to day four as observed in the MTT cell proliferation assay ( Figure1C). Additionally, CPE siRNA treatment reduced colony formation in Panc-1 cells by 62.8%, versus cells treated with scrambled (scr) siRNA ( Figure 1D and E).

**Figure 2.**
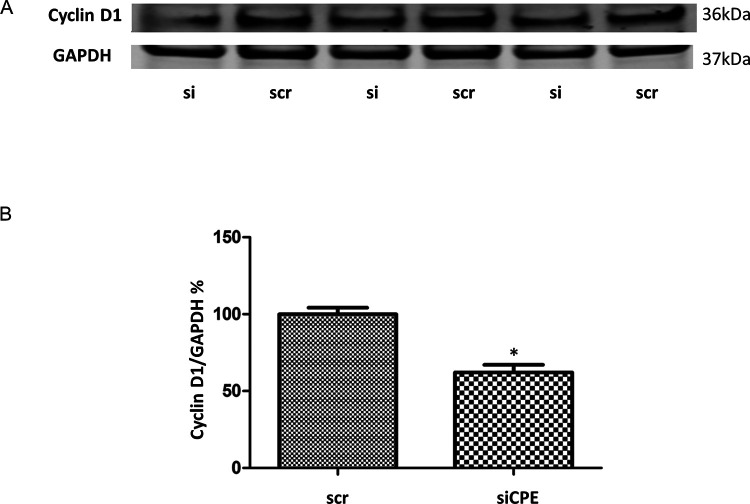
Decreased expression of Cyclin D1 in CPE siRNA treated Panc-1 cells. A. Western blot showing expression of CyclinD1 in Panc-1 cells with CPE siRNA or scrambled (scr) siRNA treatment. Full-length blots are deposited in the data repository. B. Bar graph showing reduced expression of Cyclin D1 in Panc-1 cells after CPE siRNA compared to scr siRNA treatment (n = 3, *p = 0.016). GAPDH served as loading control. Error bars denote SEM. Similar results were obtained in a second experiment.

To determine if the inhibition of proliferation of Panc-1 cells is mediated by down-regulation of Cyclin D1, a protein necessary for cell cycle progression, Western blot analysis of Panc-1 cells treated with CPE-siRNA was carried out. Figure 2A and B show that Cyclin D1 expression was significantly inhibited by 32.5% in CPE siRNA treated cells compared to scr siRNA treated cells.

### 3.2 Suppression of CPE expression inhibits migration and invasion of Panc-1 cells.

**Figure 3.**
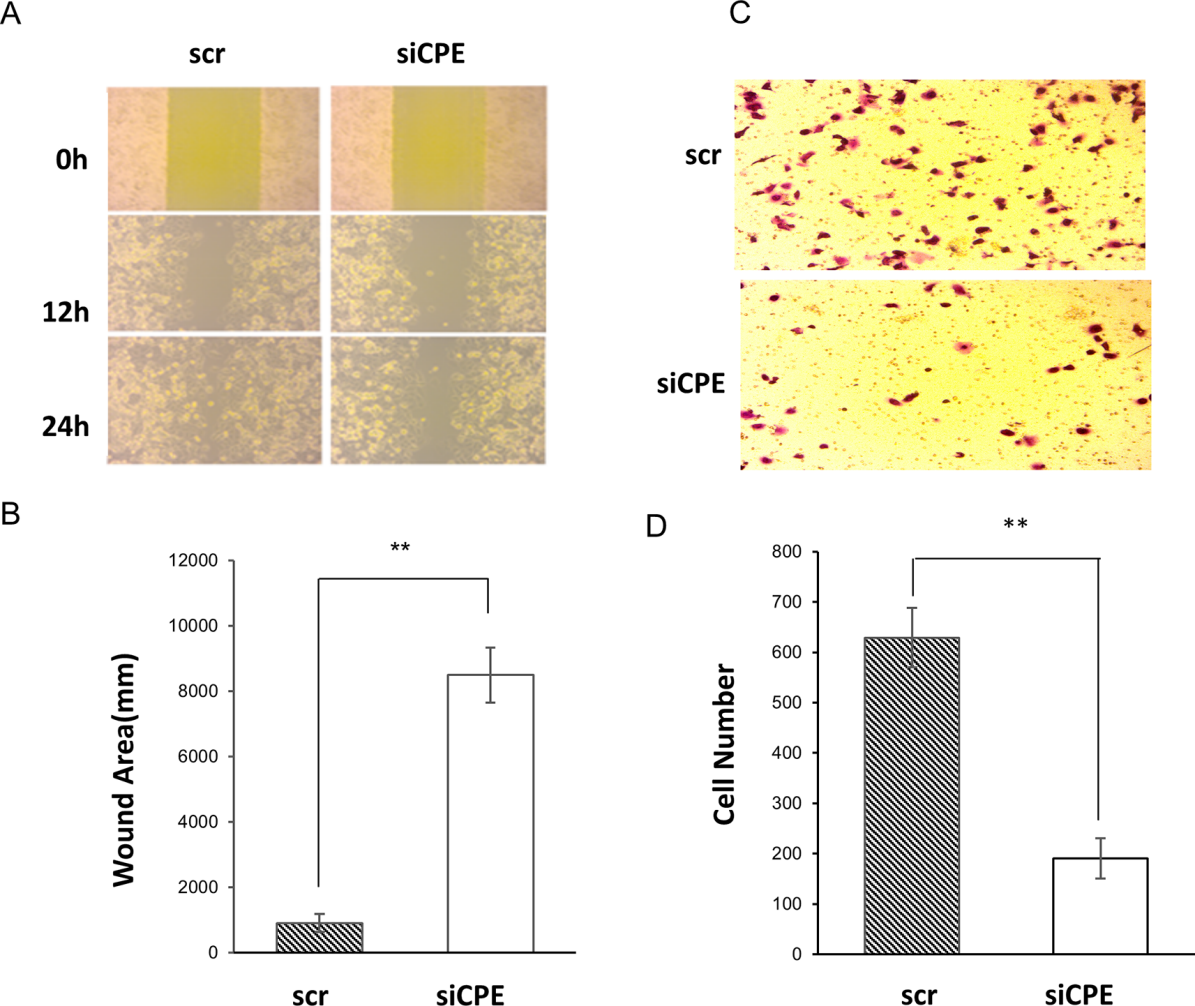
Decreased migration and invasion in CPE siRNA treated Panc-1 cells. A. Representative images of wound healing assay showing wound recovery in Panc-1 cells of CPE siRNA or scrambled siRNA treated group at 0, 12 and 24 hours. B. Bar graph showing reduced recovery, represented by larger wound area in Panc-1 cells treated with CPE siRNA (siCPE) at 24-h versus scrambled siRNA (n = 3, **p < 0.01). Representative of three experiments performed on separate days. Error bars denote SEM. C. Representative image of cells in Matrigel invasion assay of Panc-1 cells treated with CPE siRNA or scrambled siRNA (scr, control) for 24h. D. Bar graph showing reduced number of invaded Panc-1 cells after CPE siRNA compared to scr siRNA treatment (N = 3, **p < 0.01). Error bars denote SEM.

Wound healing assay was carried out to study the effect of down-regulation of CPE expression on migration of Panc-1 cells. Treatment of Panc-1 cells with CPE siRNA significantly inhibited migration of these cells. At the 24-hour time point, the wound healing area of siCPE RNA treated cells was ~ 8-fold larger than scr siRNA treated cells ( Figure 3A and B). In addition, suppression of CPE expression by CPE siRNA inhibited invasion of these cells, as revealed by the matrigel invasion assay. The number of cells which invaded across the matrigel membrane was ~three-fold greater in scr siRNA treated versus CPE siRNA treated cells ( Figure 3C and D).

## 4. Discussion

Previous studies have detected little or no WT-CPE protein present in Panc-1 and BXPC-3 pancreatic cancer cell extracts, although there was a high amount of WT-CPE mRNA in these cells. ^14^ Instead, WT-CPE was found primarily secreted into the medium in both these pancreatic cancer cell lines. ^14^ This is similar to that observed in liver cancer (HCC97H), ^10^ ovarian cancer (COAV3) ^10^ and glioblastoma cell lines. ^15^ The splice variant, 40 kD CPE-ΔN was found in the cytoplasm and the nucleus in Panc-1 cells, ^14^ similar to that found in other cancer cell lines from liver (HCC97H) and ovarian (CAOV3) cancers. ^10^ Suppression of CPE expression by CPE si-RNA which does not distinguish between WT-CPE and CPE-ΔN resulted in inhibition of proliferation of Panc-1 cells, as revealed by MTT and colony formation assay. Additionally, migration and invasion of Panc-1 cells were inhibited after CPE siRNA treatment. These findings suggest that CPE plays a significant role in promoting pancreatic tumor growth and metastasis. This conclusion corroborates with another study showing that down-regulation of CPE expression in the BXPC-3 pancreatic tumor cell line inhibited proliferation and invasion *in vitro*. ^16^ Moreover, inoculation of these CPE-siRNA treated cells into null mice, prevented pancreatic tumor formation *in vivo*. ^16^ Hence loss of function studies from two pancreatic cancer cell lines (Panc-1 and BX-PC3) support the importance of CPE in promoting pancreatic cancer progression.

The mechanism of action of CPE on proliferation has been examined in Panc-1 cells in our study. CPE siRNA treatment of Panc-1 cells showed down-regulated expression of Cyclin D1 and decreased proliferation, indicating that CPE acts through regulation of cell cycle in these cells. This is similar to another study where silencing of CPE expression caused decreased Cyclin D1 expression, cell cycle arrest and inhibition of proliferation of osteosarcoma cells. ^17^ In BX-PC3 cells, it was suggested that CPE may exert its tumorigenic effect via NF-κB since CPE-regulated NF-κB expression, and NF-κB-siRNA inhibited invasion in these cells.

A recent bioinformatic network analysis of transcriptional and epigenetic profiles of Panc-1 cells treated with CPE-siRNA, showed differentially expressed RNAs versus control, which included mRNA, miRNA, circRNA and IncRNA, that were correlated with cancer onset and/or progression. Furthermore, the analysis showed that certain RNAs such as HUWE1, hsa-miR-6780b-5p, has_circ_0058208 and lnc-G3BP1-3:8 were found to be in central positions of the network, suggesting their importance in promoting pancreatic cancer progression on many levels. ^18^

In gain of function studies, overexpression of CPE-ΔN in Panc-1 cells increased CXCR2, a pro metastatic gene. ^14^ Likewise, in a hepatocellular carcinoma cell line, (HCC97L cells), transfection of 40 kD CPE-ΔN resulted in >3-fold increase in expression of several tumor metastasis-related genes, CCXCR2, CXCR4, and CCL12. ^10^

Taken together, secreted WT-CPE which acts extracellularly and CPE-ΔN which is translocated to the nucleus ^10, 14^ are able to regulate a large number of genes that promote proliferation and metastasis, through different pathways, in pancreatic cancer cell. Some of these mechanisms mediated by CPE may be functioning in other types of cancers as well, to promote cancer progression. ^10, 13, 14^ Our study together with others suggest that CPE is a potential novel therapeutic target for treating pancreatic cancer. Inhibiting CPE expression through delivery of CPE siRNA/shRNA into tumor cells via exosomes could represent a new therapeutic approach in suppressing pancreatic tumor growth. Indeed, it has been demonstrated that CPE-shRNA loaded HEK293 cell exosomes can inhibit proliferation when taken up by highly malignant recipient HCC97H cells. ^19^ As proof of concept, exosomes carrying *KRAS* specific siRNA injected into orthotopic pancreatic cancer mouse models have been shown to suppress tumor growth, inhibit metastasis and enhance overall survival of these animals. ^20^ Thus, future studies could focus on injecting CPE sh-RNA loaded exosomes into orthotopic pancreatic cancer mouse models to test for efficacy in improving survival of these animals.

## Data availability

### Underlying data

Harvard Dataverse: Silencing of Carboxypeptidase E expression inhibits proliferation and invasion of Panc-1 pancreatic cancer cells. https://doi.org/10.7910/DVN/TUFG9M. ^21^

This project contains the following underlying data

• Figure 1B. tab (CPE qRT-PCR data in Panc-1 cells treated with CPE siRNA (siCPE) or scrambled siRNA (scr).)
• Figure 2 source data.tab (Quantification of Western Blot of CyclinD1 in Panc-1 cells treated with siCPE or scr RNA)
• Figure 1C source data.tab (MTT assay of Panc-1 cells treated with siCPE or scr RNA)
• Figure 1D source data.tab (Quantification of the number of colonies formed in Panc-1 cells treated with siCPE or scr RNA)
• Figure 3 invasion data.tab (Quantification of Panc-1 cells treated with siCPE or scr RNA in invasion assay)
• Figure 3 wounding data.tab (Quantification of wound area formed by Panc-1 cells treated with siCPE or scr RNA)
