# Supplementary material for: Silencing of Carboxypeptidase E expression inhibits proliferation and invasion of Panc-1 pancreatic cancer cells
Source: F1000Res. 2022 Apr 28;10:489. Originally published 2021 Jun 22. [Version 2] doi: 10.12688/f1000research.53737.2 (PMC9069412; doi:10.12688/f1000research.53737.2)
Supplement: Peer review history

# Peer review: Reviewer response for version 2

**DOI:** 10.5256/f1000research.121874.r136270

**Published:** 2022-04-29

**Authors:** Vinayak C. Palve

**Affiliations:** 1 Department of Drug Discovery, H. Lee Moffitt Cancer Center, Tampa, FL, USA

I thank the authors for addressing the concerns and suggestions. 

 Best of luck.

Is the work clearly and accurately presented and does it cite the current literature?

Yes

If applicable, is the statistical analysis and its interpretation appropriate?

Partly

Are all the source data underlying the results available to ensure full reproducibility?

Yes

Is the study design appropriate and is the work technically sound?

Partly

Are the conclusions drawn adequately supported by the results?

Partly

Are sufficient details of methods and analysis provided to allow replication by others?

Yes

Reviewer Expertise:

Cancer cell biology, cell signaling, drug resistance, mechanism of acquired resistance, drug discovery, pancreatic cancer, lung cancer, molecular biology, cell biology, biochemistry, off-targets of drugs, cancer cell signaling, etc.

I confirm that I have read this submission and believe that I have an appropriate level of expertise to confirm that it is of an acceptable scientific standard.

# Peer review: Reviewer response for version 1

**DOI:** 10.5256/f1000research.57152.r92019

**Published:** 2021-09-01

**Authors:** Lata Singh

**Affiliations:** 1 Department of Ophthalmology, Gavin Herbert Eye Institute, University of California Irvine, Irvine, CA, USA; 2 Department of Pediatrics, All India Institute of Medical Sciences, New Delhi, India

- The manuscript entitled 'Silencing of Carboxypeptidase E expression inhibits proliferation and invasion of Panc-1 pancreatic cancer cells ' is well written and planned study. This is the study how silenced CPE enzyme plays an important role in tumorigenesis of pancreatic cancer.
- There are some minor concerns regarding this manuscript - Please provide the full blot image of western blotting of cyclin D1 and GAPDH. 

Is the work clearly and accurately presented and does it cite the current literature?

Yes

If applicable, is the statistical analysis and its interpretation appropriate?

Yes

Are all the source data underlying the results available to ensure full reproducibility?

Yes

Is the study design appropriate and is the work technically sound?

Yes

Are the conclusions drawn adequately supported by the results?

Yes

Are sufficient details of methods and analysis provided to allow replication by others?

Yes

Reviewer Expertise:

Oncology, Pediatrics, Ocular cancers, Immunotherapy

I confirm that I have read this submission and believe that I have an appropriate level of expertise to confirm that it is of an acceptable scientific standard.

# Response

**Published:** 2022-02-22

**Authors:** Y Peng Loh

**Affiliations:** Section on Cellular Neurobiology, National Institute of Child Health and Human Development, National Institutes of Health, USA

**Response to reviewer 2**

 Thank you for the comment. We had already provided a full blot image of western blot of cyclin D1 and GAPDH in the Harvard website where the raw data are saved and publicly available.  Please click on the link in the paper, or for your convenience here is the link below and you can see the full length gel.

https://doi.org/10.7910/DVN/TUFG9M,

# Peer review: Reviewer response for version 1

**DOI:** 10.5256/f1000research.57152.r88445

**Published:** 2021-07-08

**Authors:** Vinayak C. Palve

**Affiliations:** 1 Department of Drug Discovery, H. Lee Moffitt Cancer Center, Tampa, FL, USA

In the present manuscript, the author's aims to understand the role of Carboxypeptidase E  in regulating proliferation, migration and invasion to promote pancreatic cancer tumorigenesis. The authors present some data but I believe it needs further evidence to support the conclusion made in the current manuscript. I would recommend implementing the following suggestions would improve the overall quality of the manuscript. 

**Major comments: **

1. The authors have used only one pancreatic cell line (Panc-1) throughout the manuscript. Generating data for most of the experiment in 2-3 pancreatic cell lines would strengthen the findings.

**Minor comments:**

1. It would be interesting to describe, how the different variants of CPE works?
2. Western blots in Figure 2A need to be cleaner. The GAPDH blot is not cropped well. I suggest to add better quality of blots and load the lysates taking control at first followed by CPEsi RNA.
3. Please provide the good quality and resolution of images (especially for Figure 3). 
4. The authors can blot for downstream targets of Cyclin D1 to address how depletion of Cyclin D1 helps the pancreatic cells to stop proliferation in a more mechanistic approach. 

I believe addressing these comments shall help improve the quality of the manuscript. All the best.

Is the work clearly and accurately presented and does it cite the current literature?

Yes

If applicable, is the statistical analysis and its interpretation appropriate?

Partly

Are all the source data underlying the results available to ensure full reproducibility?

Yes

Is the study design appropriate and is the work technically sound?

Partly

Are the conclusions drawn adequately supported by the results?

Partly

Are sufficient details of methods and analysis provided to allow replication by others?

Yes

Reviewer Expertise:

Cancer cell biology, cell signaling, drug resistance, mechanism of acquired resistance, drug discovery, pancreatic cancer, lung cancer, molecular biology, cell biology, biochemistry, off-targets of drugs, cancer cell signaling, etc.

I confirm that I have read this submission and believe that I have an appropriate level of expertise to confirm that it is of an acceptable scientific standard, however I have significant reservations, as outlined above.

# Response

**Published:** 2022-02-22

**Authors:** Y Peng Loh

**Affiliations:** Section on Cellular Neurobiology, National Institute of Child Health and Human Development, National Institutes of Health, USA

**Major comments **

1. We agree with the reviewer that generally, studies should be done on 2 cell lines .  However since there is a publication already with similar results using the BXPC3 pancreatic cell line , we feel that our findings are supported by another pancreatic cell line already and there was no need to do it on an additional cell line.

**Minor comments:**

1. We have published a paper (ref14)  showing that CPE -WT  promotes proliferation while the CPE-DN promotes both proliferation and invasion via up-regulation of CXCR2 , a pro-metastatic gene in Panc-1 cells. This was reported in the Discussion section.

**2,3. **Thanks for these comments. We have provided cleaner Western Blots and have asked F1000research to help exchange the figures in the first version for the new figures.   

**4.   **We agree that additional work to investigate the mechanism of how downstream of depleting    Cyclin D1 could stop proliferation is a good idea. However, to blot for and identify potential downstream targets of Cyclin D that control proliferation via signaling that regulates cell cycle progression requires extensive work and beyond the scope of this paper.  

 We hope that this reviewer can now approve this paper. Thank you.
